# Experimental Investigation of Multi-mode Fiber Laser Cutting of Cement Mortar

**DOI:** 10.3390/ma11020278

**Published:** 2018-02-10

**Authors:** Dongkyoung Lee, Sukhoon Pyo

**Affiliations:** 1 Department of Mechanical and Automotive Engineering, Kongju National University, Cheonan-si 31080, Chungcheongnam-do, Korea; ldkkinka@kongju.ac.kr; 2 Korea Railroad Research Institute, Uiwang-si 16105, Gyeonggi-do, Korea

**Keywords:** cement mortar, heat treatment, multi-mode fiber laser, chemical analysis

## Abstract

This study successfully applied multi-mode laser cutting with the variation of the laser cutting speed to cement mortar for the first time. The effects of the amount of silica sand in the cement mortar on laser cutting are tested and analyzed. The kerf width and penetration depth of the specimens after laser cutting are investigated. As the laser cutting speed increases, the penetration depth decreases for both cement paste and cement mortar, whereas the kerf width becomes saturated and increases, respectively, for cement paste and cement mortar. Cross sections of the specimens are compared with illustrations. Top-view images of the cement mortar with indicators of the physical characteristics, such as re-solidification, burning, and cracks are examined, and the possible causes of these characteristics are explained. The optical absorption rates of cement-based materials are quantified at wide ranges of wavelength to compare the absorption rates in accordance with the materials compositions. The chemical composition variation before and after laser cutting is also compared by EDX (Energy Dispersive X-Ray) analysis. In addition to these observations, material removal mechanisms for cement mortar are proposed.

## 1. Introduction

Laser-aided manufacturing (LAM) technologies have been used in numerous applications not only for metal alloys [1], but also for composites materials [2], bio-tissues [3], etc. with their various advantages over conventional method, e.g., a higher precision level, faster speed, and non-contact method. Among LAM technologies, laser cutting has been widely used in many applications, such as lithium-ion batteries [4,5,6,7,8], electronic devices [9,10], automotive [11], ship building, aircraft industries [12,13]. Despite its popularity and wide uses for various materials, there are relatively few applications in the cement-based construction materials. Laser irradiation techniques have been tested in this applications to remove degraded parts of a concrete structure, such as railways tunnel concrete lining [14] and radioactive-contaminated concrete [15]. To remove the surface material of concrete using a high-power laser beam, laser scabbling of concrete has been studied [15,16,17]. Key factors affecting the laser scabbling process were found. Moreover, studies of the relationships among laser interaction time, volume removal, and surface temperatures for different compositions were presented to identify the effect of concrete composition on the laser scabbling behavior. However, these studies used a low laser power density (176.83 W/cm^2^; laser power of 5 kW and spot diameter of 60 mm) and were very limited to the removal of surface layers. To remove more volume of concrete, a higher laser beam density is required. Since a high-power laser beam (10 kW) is commercially available and can be focused on very small spots up to 10 μm by manipulating the optics, laser cutting can be also applied to remove large volumes of cement-based construction materials. 

This research focuses on the laser cutting of cement mortar in order to systematically find adequate parameters of the laser beam. Furthermore, the material removal mechanisms during the interaction between the laser and the cement mortar are studied. The material compositions and the experimental setup used in this research are firstly described. Then, the effects of compositional variations of the cement mortar after laser cutting are discussed. Furthermore, geometrical parameters such as the kerf width and the penetration depth of the workpiece are characterized depending on the laser cutting speed. The optical absorption rates of cement-based materials are evaluated to describe the interactions between the laser and the tested materials. The chemical reaction of the cementitious composites is also evaluated using the Energy Dispersive X-Ray (EDX) analysis. Finally, the important findings of this study are summarized.

## 2. Materials and Mix Design

Three types of cement-based materials with a thickness of about 4 mm were prepared. The materials’ composition are shown in Table 1. Type I Ordinary Portland cement (OPC), water, and silica sand with a median diameter of 0.53 mm were used. Each series name was designated by the amount of silica sand in order to simplify the discussion of the results. LP stands for cement paste mixed with OPC and water, whereas LM stands for cement mortar mixed with OPC, water, and silica sand. In order to identify the effects of the amount of silica sand in the cement mortar, two different LM series were also prepared, where LM1 and LM series have the same amount of silica sand and 1.5 times the amount of silica sand compared to OPC, respectively. 

A laboratory planetary mixer was used to prepare all mixtures. Cement, water, and silica sand, if any, were mixed together. Once the mixture started to show adequate consistency, the wet mixture was poured into 50 mm cubic molds for compression tests and into 50 × 50 × 4 mm^3^ molds for laser tests. The casted specimens were covered with plastic sheets and stored at room temperature for 24 h prior to demolding. The specimens were then cured in a water tank at 23 °C. The compressive strength was evaluated in dry conditions at the age of 28 days, after 24 h of drying in the laboratory environment. The evaluated compressive strength values were averaged using at least three specimens for each series, and the averaged compressive strength are listed in Table 1. Laser cutting tests were also carried out in dry conditions after at least 28 days of curing. The specimens for the evaluation of the absorption rate were also prepared with a size of 15 (Length) × 15 (Width) × 10 (Height) mm^3^ on top of a silicon wafer. Three types of cement-based materials with the same OPC–sand proportions as in Table 1 with a water–cement ratio of 0.4 were prepared with the aim of maintaining dimensional stability by reducing free water within the matrix.

## 3. Experiment

**Figure 1:**
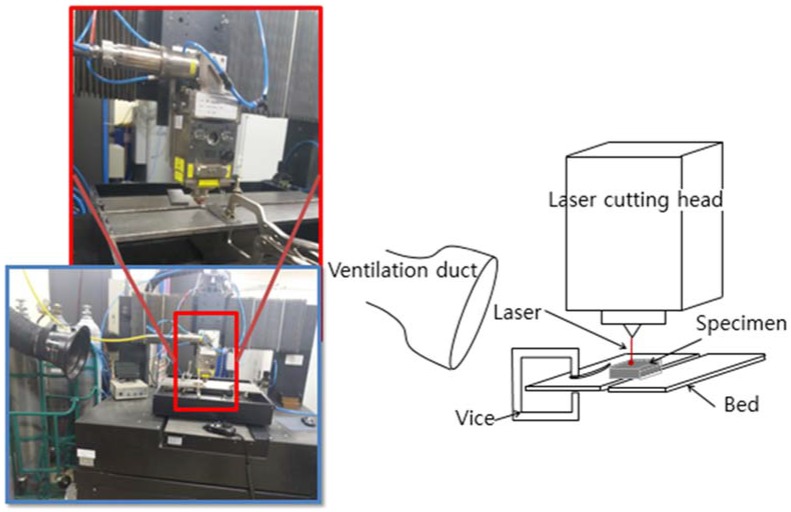
Experiment setup.

The experimental setup used in this research is shown in Figure 1. A multi-mode continuous fiber laser (IPG YLS-10000, IPG photonics, Oxford, MA, USA) is used with a laser beam diameter of 150 μm at focus. Its maximum available laser power is 10 kW, and the wavelength of the laser is 1070 nm. A workpiece is placed on the bed. The bed consists of two pieces with a 65 mm gap between the beds to provide enough space where the processed material can be removed. The laser beam is vertically irradiated on the workpieces fixed with a vice, and the laser head is moving while cutting. To collect the dust created during the experiment, a ventilation duct is placed in the back of the laser cutting direction. Only the laser cutting speed is set to a control parameter, beside the material composition, to make the experiments simple, since the application of laser cutting on cement mortar is attempted in this research for the first time. The laser power used in this study is 1 kW.

The laser cutting speed is increased from 4 m/min to 14 m/min at intervals of 2 m/min. N_2_ assistant gas is used with a pressure of 7 bar. The parameters, such as the laser cutting speed and line energy, used in the experiments are shown in Table 2. The line energy is defined by dividing the laser power by the laser spot size and laser cutting speed, as follows:

(1) $$E_{line}=\frac{P_{laser}}{V_{s}\times A}\;\left(J/m^{3}\right),$$

where $P_{laser}$ is the laser power, $V_{s}$ is the scanning speed, and A is the laser spot size. The line energy is an important parameter in the area of laser cutting to understand material removal mechanisms and evaluate the laser cutting efficiency.

In addition to the laser cutting experiments, the absorption rates of three types of cement-based materials are evaluated to estimate the interaction mechanism between the laser and the tested materials. To obtain the absorption rates, the transmission and reflection rates are first measured by UV–VIR–NR spectrophotometry (SolidSpec-3700, Shimadzu, Kyoto, Japan). The absorption rates are calculated by subtracting the transmission and reflection rates from 100% of input beam.

## 4. Results and Discussion

### 4.1. Visual Observation

**Figure 2:**
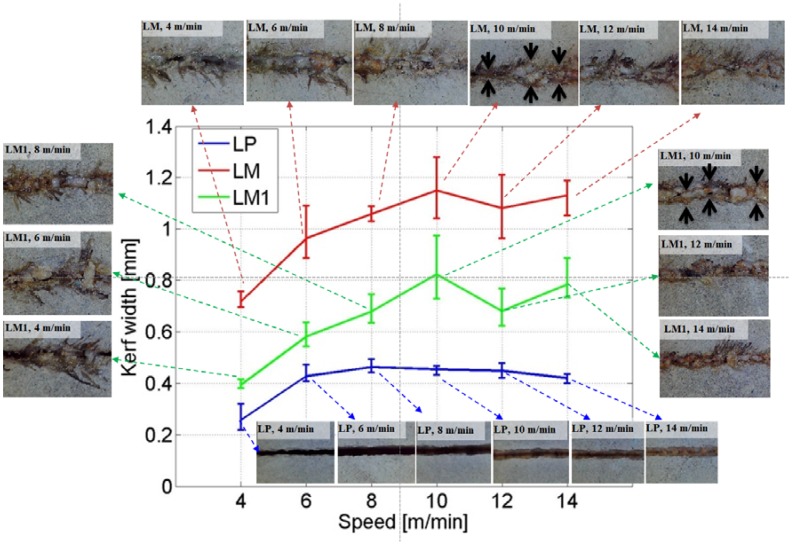
Relationship between kerf width and laser speed and related images.

**Figure 3:**
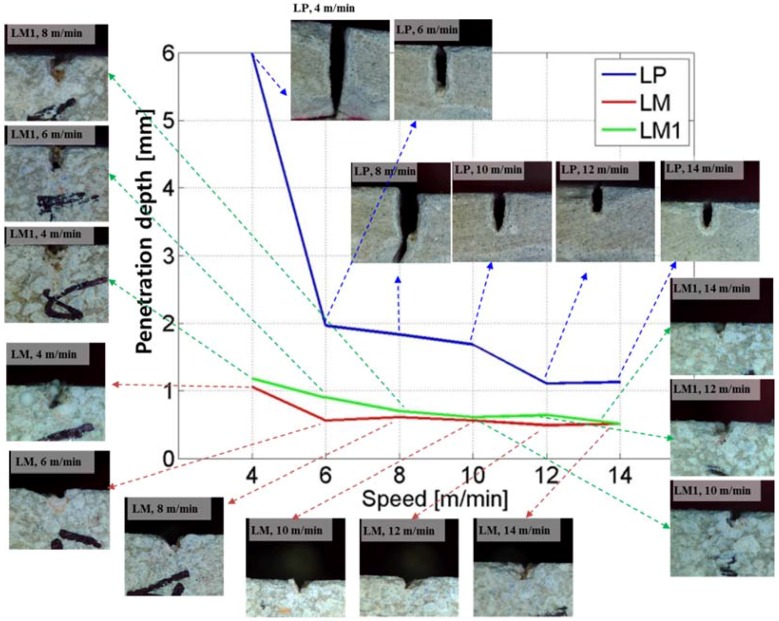
Relationship between penetration depth and laser speed and related images.

The kerf width and penetration depth of laser cutting are set to characteristic variables. Figure 2 and Figure 3 show the variation of the kerf width and penetration depth, respectively, depending on the laser scanning speed and materials used. Even though the kerf width of the LP series can be clearly observed, the kerf width of LM and LM1 is not as clear as that of the LP series. The kerf width of LM and LM1 is measured at the boarder of the material removal region including the re-solidification region and the unprocessed region, while the crack is ignored for the width measurement. The measured points are shown with a black arrow in Figure 2.

**Figure 4:**
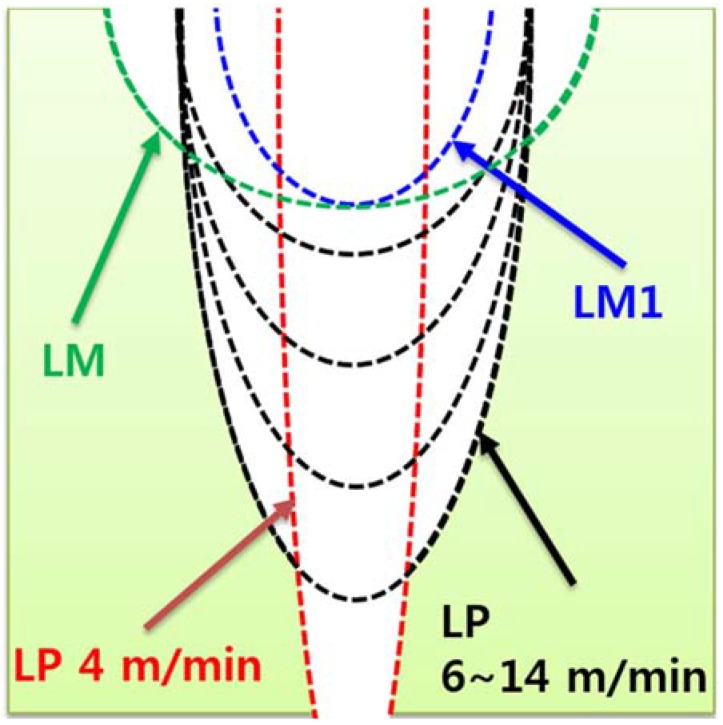
Characteristics of laser cutting of the cementitious composites.

For the LP series, the average kerf width varies between 0.42 and 0.46 mm, which is almost consistent, with laser cutting speeds of 6–14 m/min. When the laser cutting speed is 4 m/min, the average kerf width is 0.27 mm. All the workpieces show a partial penetration, except for one, the LP workpiece to which the laser cutting speed of 4 m/min is applied, as seen in Figure 3. On the basis of these observations, the characteristics of laser cutting of the LP series can be illustrated as Figure 4. When the hole is partially penetrated, the penetration depth increases from 1.13 to 1.97 mm as the laser cutting speed decreases, while no significant variation is observed for the kerf width.

Therefore, it can be hypothesized that most of the laser energy is transferred downward instead of to a transverse direction for the LP series, since there is less significant variation of the kerf width. In addition, the energy transfer mechanism in the transverse direction is different in the case of metal laser cutting, as reported in the literature [8,18,19,20,21]. In the case of metal, both the hole depth and the hole width increase gradually as the laser cutting speed decreases. Hence, the material removal mechanism during laser cutting of the LP series may be explained differently with respect to the case of metal laser cutting.

**Figure 5:**
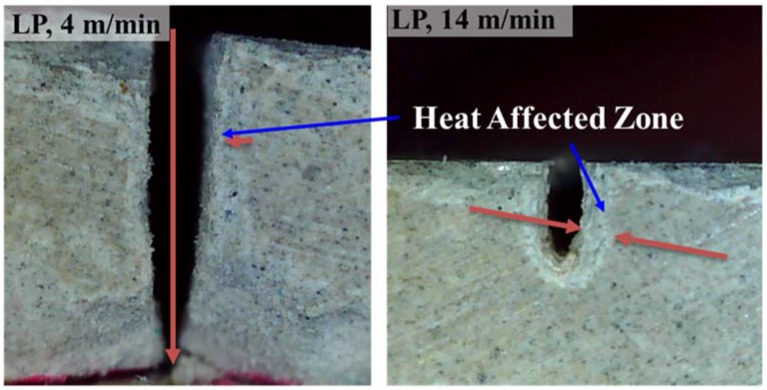
Heat-Affected Zone (HAZ) on the LP series.

As for the line energy, a line energy of 8.49 × 10^12^ J/m^3^ is enough to fully cut the LP series with the thickness of 4 mm. However, the other experimental cases, having a line energy lower than 8.49 × 10^12^ J/m^3^, show only a partial penetration. From this investigation, it can be concluded that the laser cutting threshold for the LP series is between 4 m/min and 6 m/m, or 8.49 × 10^12^ J/m^3^ and 5.66 × 10^12^ J/m^3^. A cross section of the LP series is shown in Figure 3. In all cases, a Heat-Affected Zone (HAZ) can be clearly observed, as seen in Figure 5. The HAZ varies between 0.40 and 0.51 mm and no significant variation is observed for the given laser parameters. This evidence confirms that the material removal mechanism during the laser cutting of the LP series is different compared to the metal cases. 

In contrast with the LP series, the LM series shows a transverse heat transfer direction. As the laser cutting speed increases, the kerf width increases, and the penetration depth decreases gradually. Compared to the LP series, the LM and LM1 series show a larger kerf width and a shallower penetration depth. While the penetration depths of the LM and LM1 series are in a similar range, which is between 0.5 and 1 mm, the kerf width shows clear differences among the LP, LM, and LM1 series. The penetration depth of the LP series is almost twice as much as that of the LM and LM1 series. However, the kerf width of the LM series is almost double the kerf width of the LM1 series, while an almost similar trend is observed for the penetration depth. In addition, the kerf width of the LM1 series is almost double the kerf width of the LP series. Two interesting facts can be derived from this comparison. First, the effect of silica sand on cement mortar during laser cutting is clearly seen. Second, the material removal mechanism of the LM and LM1 series may be different from that of the LP series.

In addition to the above-mentioned comparison, the characteristics of laser cutting in the LM and LM1 series can be observed. For the LM and LM1 series, the kerf width and the penetration depth saturate when the laser scanning speed becomes higher than 10 m/min. The kerf width increases as the ratio of the silica sand in the cement mortar increases. However, the penetration depth decreases as the ratio of silica sand increases. This may be because an interaction between the silica sand and the laser would provide more heat to the transverse direction. Hence, it can be hypothesized that the silica sands play a major role in changing the direction of the heat transfer. 

**Figure 6:**
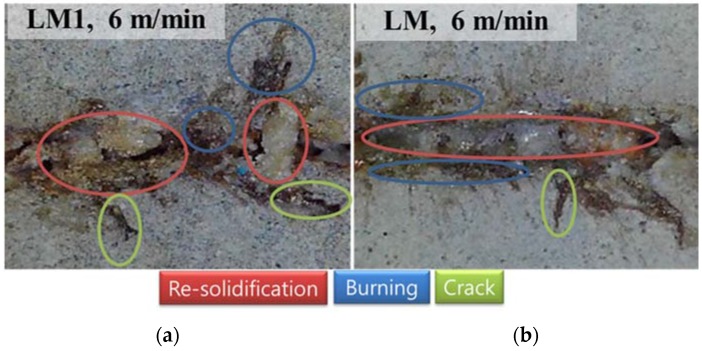
Top view of (**a**) LMI and (**b**) LM with indicators of the physical characteristics.

Furthermore, an uneven kerf width is observed in the images of the LM and LM1 series, while an even kerf width is clearly observable in the images of the LP series. In this uneven kerf width, several phenomena can be observed. The top view images with indicators of the physical characteristics are shown in Figure 6. From this figure, three physical characteristics, i.e., re-solidification, burning, and cracks can be seen. Re-solidification is shown in the middle of the kerf width. The re-solidification region shows a smoother surface than the other regions, with a fine porous appearance. From this observation, it can be concluded that the silica sand may be melted and re-solidified so that it forms smoother and bigger grains. Around this re-solidification region, burning is also observed. Burning can be presumed because the color is changed from gray to black. Lawrence and Li [22,23] expressed this burning as a ‘dramatic color change’ and explained the change from the chemical composition point of view. Since the black color, which is determined by the Fe^3+^/Fe^2+^ ion ratio, is observed, it can be inferred that both phases are present within the composition [22,23,24]. Finally, a crack is observed on the region which is a little farther than the re-solidification region. It can be assumed that the high pressure applied during laser cutting may be exhausted through this region.

### 4.2. EDX Analysis

**Figure 7:**
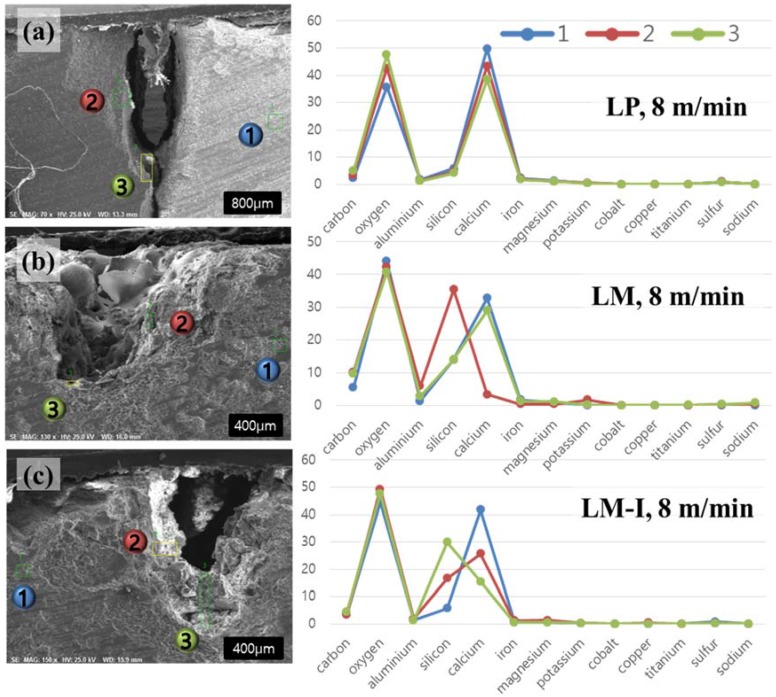
Composition changes with a laser scanning speed of 8 m/min (**a**) LP; (**b**) LM; (**c**) LM1.

EDX analysis is carried out to observe the variation of the chemical compositions of cement-based materials after the interaction with the laser. The images and graphs of this EDX analysis with a laser speed of 8 m/min are shown in Figure 7. Three points per each case are chosen for this analysis. The first point, where laser cutting is not affected, is chosen and indicated with the blue color. Two additional points are selected, where laser cutting is directly applied and indicated with the green and red colors. The compositions by materials and points are tabulated in Table 3, with a laser speed of 8 m/min. For the LP series, while a little variation of composition is observed, no significant change is seen. Before applying the laser cutting, which is the first point, calcium, silicon, and oxygen show 49.80%, 5.85%, and 35.61% of their weight percent, respectively. The weight percent of calcium, silicon, and oxygen changes to 38.41%, 4.07%, and 47.49%, respectively, after laser cutting. From this composition changes, it can be inferred that silicon and calcium in the cement hydration products (e.g., calcium silicate hydrate) change to silicon dioxide (SiO_2_) and calcium oxide (CaO) because of the interaction between the laser and the LP specimen. Even though the weight percent of calcium and oxygen is changed, these elements are still a major part of the LP specimen, and the graph trend is still the same as observed in the first point. 

Different to the LP case, the LM and LM1 series show significant changes of the weight percent of silicon and calcium. For the LM and LM1 series, calcium is abundant before applying the laser cutting. However, after applying the laser cutting, the weight percent of calcium decreases, while that of silicon increases. This indicates that the silica sand is melted and aggregated so that the bigger body is formed as shown in Figure 6, while some portions of calcium are removed. For the LM series, the most significant change regards the weight percent of silicon and calcium. The weight percent of calcium decreases from 32.75% to 3.32% between point 1 and point 2. On the other hand, the weight percent of silicon increases from 13.95% to 35.43% between point 1 and point 2. Furthermore, point 1 and point 3 show the same graph trend as shown in Figure 7b. For the LM1 series, the most significant change is also seen in the weight percent of silicon and calcium. The weight percent of silicon and calcium at point 1 is 5.74% and 41.62%, respectively. As observed from the LM series, point 2 and point 3 of the LM1 also show that the weight percent of silicon increases and that of calcium decreases. However, the amount of variation is not as significant as in the LM case. The weight percent of silicon is smaller than that of calcium at point 2 of the LM1 specimen, while the weight percent of silicon is greater than that of calcium at point 1 of LM1 series. 

### 4.3. Material Removal Mechanism during Laser Cutting

As mentioned in the earlier section, the LP series shows a very good cutting quality. It is interesting to note that laser cutting is successfully applicable to a cement paste in which only cement and water are mixed. Since it shows a very clean cut surface, it is expected that the laser cutting mechanism of cement-based materials may be different to that of metal, where the main mechanisms of material removal are melting and evaporation, as proposed by many studies [8,18,19,20,21].

**Figure 8:**
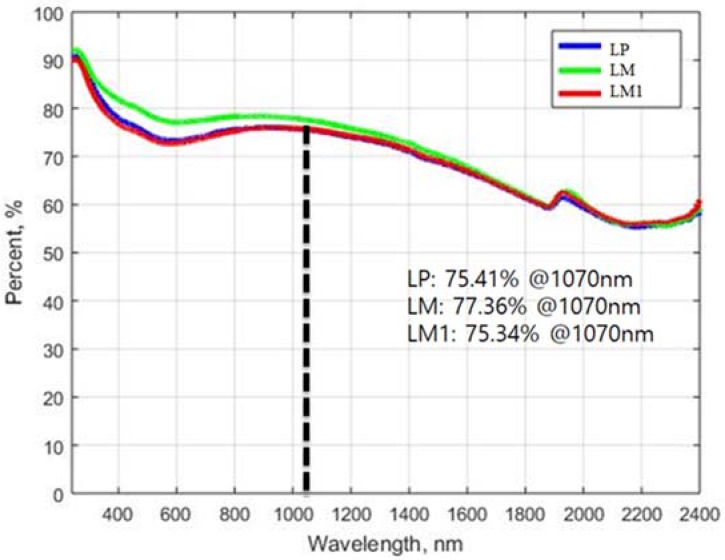
Comparison of the absorption rates of LP, LM, and LM1 according to various wavelength.

Before directly investigating the material removal mechanism, the absorption rates on the flat surface are obtained and all measured samples are compared. The absorption rates of LP, LM, and LM1 are shown in Figure 8 over wavelengths ranging from 200 to 2400 nm. The absorption rates at the wavelength of 1070 nm, which is the same wavelength as that of the used laser in this research, are 75.41%, 77.36%, and 75.34% for LP, LM, and LM1, respectively. The absorption rates are almost similar to each other. This might be explained by the fact that cement hydration products are abundant and fully coat the surface of the silica sands in the cement mortar series. On the basis of the absorption rate comparisons, one may expect that the laser cutting efficiency of cement mortar is high because of the high absorption rate. In addition, it is found that the laser cutting mechanism is strongly related to the thermal and/or optical characteristics of the contents. Because similar trends of absorption rates regardless of the contents of silica sands are observed, one can conclude that the laser cutting mechanism is strongly related to the thermal and/or optical characteristics of the individual constituents rather than to the absorption rates of the composites. Therefore, the cutting mechanism needs to focus on the material composition inside the material.

**Figure 9:**
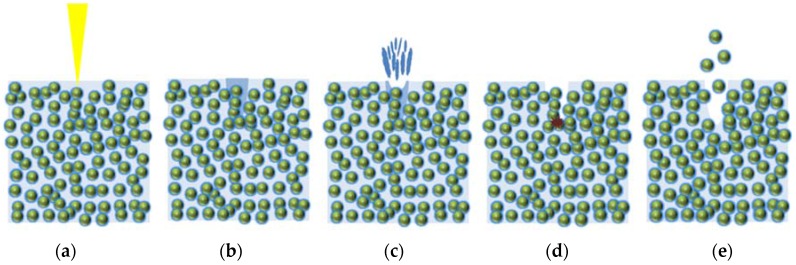
Mechanism of laser cutting of the LP series. (**a**) the laser is irradiated; (**b**) the heat provided by the laser increases the temperature of the cement hydration products; (**c**) water inside the cement hydration products can reach its evaporation temperature; (**d**) some cement hydration products decompose and some chemical bonds are weakened, and high pressure forms inside the specimen; (**e**) the decomposed cement hydration products are blown away by N_2_ assistant gas.

The LP series is composed of OPC, with particle sizes are around 1–50 μm, and water, so that the bonding strength between cement hydration products is lower than for the atomic bonding of metals. Hence, it is reasonable to propose a laser cutting mechanism of cement-based materials based on the following assumption. The description of the laser cutting mechanism of the LP is shown in Figure 9. First, the heat provided by the laser increases the temperature of the cement hydration products (see Figure 9b). If heat is provided to the specimen, water inside the cement hydration products can reach its evaporation temperature, as depicted in Figure 9c. While water is evaporating, some cement hydration products decompose and some chemical bonds are weakened, and high pressure forms inside the specimen (see Figure 9d). Furthermore, N_2_ assistant gas is blown into the interaction zone at a pressure of 7 bar during laser cutting. Because of this high pressure, the decomposed cement hydration products are blown away easily (see Figure 9e). During the process of material removal of the LP series, the composition of the cement hydration products may not be significantly changed since the evaporation temperature of water is much lower than the melting temperature of the cement hydration products [25]. As measured from Figure 8, 75.41% and 24.59% of the laser beam is absorbed and reflected, respectively. Thus, multiple reflections occur continuously until the reflected laser beam is dissipated or escapes from the penetration hole. Once the material is removed and the penetration hole is created, the bundle of the laser beam is gathered at the tip of the penetration hole after the multiple reflections. Therefore, laser energy gathering as a result of the multiple reflections and material removal from the tip of the penetration hole occur sequentially and repeatedly, so that most of the heat energy is transferred downward.

**Figure 10:**
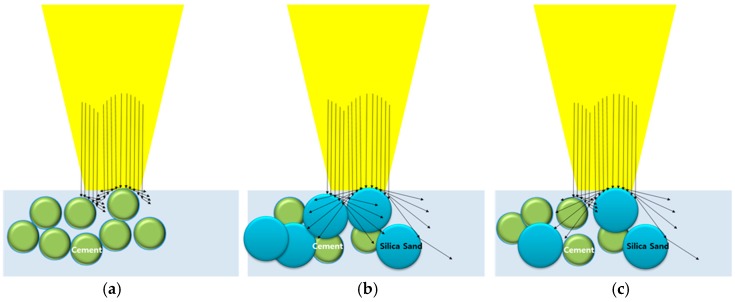
Comparison of the material removal mechanisms of (**a**) LP; (**b**) LM; (**c**) LM1.

In contrast to the LP series, the LM and LM1 series show unfavorable cutting qualities, and almost no material is penetrated compared to the LP series. Hence, another material removal mechanism need to be introduced to describe these cement mortar series. The removal mechanism of the cement mortar may be explained by two physical properties: absorption rate and melting temperature. The proposed comparison of the material removal mechanisms of the LP, LM, and LM1 series is shown in Figure 10. In this figure, the arrow indicates the reflection rates. Thus, if the arrow is long, there is high reflection and low absorption of the laser beam. Unfortunately, no data about the individual absorption rates of silica sand and cement, which is the powder form, is available. The absorption rate of silica sand may be very low, so that most of the laser beam is reflected. Therefore, the reflected laser beam is not directed to the atmospheric area but is directed sideways where other silica sand is present. If the side area contains silica sand, then again most of the laser beam is reflected and redirected sideways, and so on. Therefore, the LM and LM1 specimens have a wider kerf width than the LP specimens. On the other hand, the absorption rate of cement is relatively high so that most of the laser beam is absorbed by the cement. In addition to the absorption rate, the melting temperature also affects to the material removal mechanism of the cement mortar. The melting temperatures of cement hydration products are much lower compared to those of their solid counterpart, i.e., silica sand, since cement hydration products are relatively easily decomposed because of the evaporation of the water inside the chemical products. Meanwhile, silica sands, having a crystalline structure, have a different interaction mechanism with the laser, with a transfer of the thermal energy to the adjacent materials. 

## 5. Conclusions

This experimental study investigated the applicability of the laser cutting technique using a multi-mode continuous fiber laser to cement-based materials. The parameters tested in this research were three material compositions with different amounts of silica sand, and six laser cutting speeds, from 4 m/min. to 14 m/min. By examining the surfaces and cross sections of the specimens, the kerf width and penetration depth of all specimens were investigated. In addition, EDX analysis and absorption rates determination were performed to explain the material removal mechanisms during laser cutting. The key observations and findings of this study can be summarized as follows:

1. It is found that the line energy of 8.49 × 10^12^ J/m^3^ is enough to fully cut a cement paste with the thickness of 4 mm. Furthermore, the material removal mechanism during laser cutting of cthe ement paste is found to be different to the case of laser cutting of metals.
2. The amount of silica sand in the cement mortar leads to significant differences in laser cutting quality and characteristics. The cement paste showed a relatively even kerf width and a deeper penetration depth, whereas the cement mortar showed a wider kerf width and a shallower penetration depth under the same amount of laser energy. This phenomenon could be explained by the fact that the silica sand would contribute to changing the direction of the heat transfer as a result of the interaction between the silica sand and the laser.
3. The heat-affected zone of the LP can be clearly identified in the cross section of all specimens, and varies between 0.4 and 0.51 mm; no significant variation within the given laser parameters is found.
4. The presence of silica sand leads cement mortar specimens to additional physical changes such as resolidification, burning, and cracks under laser interaction. Because of the high pressure induced during laser cutting, cracks may develop in small regions farther from the re-solidification region. The chemical composition changes after laser interaction can be found in the EDX analysis, which indicates that silicon and calcium in the cement hydration products decompose into silicon dioxide and calcium oxide in the cement paste specimens. On the other hand, in the case of the cement mortar, silica sand is formed in a bigger structurer after laser interaction, and some portions of calcium are removed.

## Figures and Tables

**Table 1:** Mix design of the tested cementitious composites (proportions by weight).

Series	Cement	Water	Silica Sand	Compressive Strength (MPa)
LP	1	0.5	-	56.1
LM	1	0.5	1.5	65.7
LM1	1	0.5	1.0	66.4

**Table 2:** Laser parameters used in the experiments.

Cases	Speed (m/min)	Line Energy (J/m^3^)
1	14	2.43 × 10^11^
2	12	2.83 × 10^11^
3	10	3.40 × 10^11^
4	8	4.24 × 10^11^
5	6	5.66 × 10^12^
6	4	8.49 × 10^12^

**Table 3:** Composition (weight %) by materials and points.

Series	Points	Total (%)	C	O	Al	Si	Ca	Fe	Mg	K	Cu	Ti	S	Na
LP	1	100	2.20	35.6	1.68	5.85	49.8	2.22	1.26	0.41	0	0	0.97	0
	2	100	3.46	42.6	1.33	4.70	43.4	2.04	1.05	0.45	0	0	0.90	0
	3	100	4.86	47.5	1.22	4.07	38.4	1.73	1.04	0.42	0	0	0.76	0
LM	1	100	5.45	44.0	1.36	14.0	32.8	1.60	0.89	0	0	0	0	0
	2	99.99	10.2	42.4	5.83	35.4	3.32	0.29	0.28	1.63	0	0	0.24	0.35
	3	100	9.80	40.8	2.80	14.0	29.0	1.20	1.12	0.11	0	0.08	0.30	0.79
LM1	1	99.99	3.85	44.4	1.38	5.74	41.6	1.37	0.71	0.15	0	0	0.74	0
	2	99.99	3.44	49.2	1.83	16.7	25.6	1.16	1.27	0.19	0.32	0	0.28	0
	3	100	4.55	47.6	1.17	29.9	15.4	0.46	0.47	0.20	0	0	0.21	0

## References

[1] Cao X., Jahazi M., Immarigeon J.P., Wallace W. (2006). A review of laser welding techniques for magnesium alloys. J. Mater. Process. Technol..

[2] Dubey A.K., Yadava V. (2008). Laser beam machining—A review. Int. J. Mach. Tool. Manu..

[3] Ansari M.A., Erfanzadeh M., Mohajerani E. (2013). Mechanisms of laser-tissue interaction: II. Tissue thermal properties. J. Lasers Med. Sci..

[4] Lee D., Patwa R., Herfurth H., Mazumder J. (2016). Parameter optimization for high speed remote laser cutting of electrodes for lithium-ion batteries. J. Laser Appl..

[5] Lee D., Mazumder J. (2016). Effects of laser beam spatial distribution on laser-material interaction. J. Laser Appl..

[6] Lee D., Patwa R., Herfurth H., Mazumder J. (2016). Three dimensional simulation of high speed remote laser cutting of cathode for lithium-ion batteries. J. Laser Appl..

[7] Lee D., Patwa R., Herfurth H., Mazumder J. (2013). High speed remote laser cutting of electrodes for lithium-ion batteries: Anode. J. Power Sources.

[8] Lee D., Mazumder J. Numerical Studies of Laser Cutting of an Anode for Lithium-ion Batteries. Proceedings of the International Congress on Applications of Lasers & Electro–Optics.

[9] Lee D. (2017). Experimental investigation of laser spot welding of Ni and Au-Sn-Ni alloy. J. Weld. Jt..

[10] Lee D., Cho J., Kim C.H., Lee S.H. (2017). Application of laser spot cutting on spring contact probe for semiconductor package inspection. Opt. Laser Technol..

[11] Hong K.M., Shin Y.C. (2017). Prospects of laser welding technology in the automotive industry: A review. J. Mater. Process. Technol..

[12] Weng F., Chen C., Yu H. (2014). Research status of laser cladding on titanium and its alloys: A review. Mater. Des..

[13] Zhang K.R., Zhang J.X. (2009). Numerical simulation for keyhole profile and their effect of TC4 titanium alloy during laser welding. Rare Met. Mat. Eng..

[14] Long N.P., Daido H., Yamada T., Nishimura A., Hasegawa N., Kawachi T. (2017). Experimental characterization of concrete removal by high-power quasicontinuous wave fiber laser irradiation. J. Laser Appl..

[15] Peach B., Petkovski M., Blackburn J., Engelberg D.L. (2016). Laser scabbling of mortars. Constr. Build. Mater..

[16] Peach B., Petkovski M., Blackburn J., Engelberg D. (2016). The effect of concrete composition on laser scabbling. Constr. Build. Mater..

[17] Peach B., Petkovski M., Blackburn J., Engelberg D. (2015). An experimental investigation of laser scabbling of concrete. Constr. Build. Mater..

[18] Ki H., Mohanty P.S., Mazumder J. (2002). Multiple reflection and its influence on keyhole evolution. J. Laser Appl..

[19] Ki H., Mohanty P.S., Mazumder J. (2002). Modeling of laser keyhole welding: Part I. Mathematical modeling, numerical methodology, role of recoil pressure, multiple reflections, and free surface evolution. Metall. Mater. Trans. A.

[20] Ki H., Mohanty P.S., Mazumder J. (2002). Modeling of laser keyhole welding: Part II. Simulation of keyhole evolution, velocity, temperature profile, and experimental verification. Metall. Mater. Trans. A.

[21] Ki H., Mohanty P.S., Mazumder J. (2001). Modelling of high-density laser-material interaction using fast level set method. J. Phys. D Appl. Phys..

[22] Lawrence J., Li L. (1999). Surface glazing of concrete using a 2.5 kW high power diode laser and the effects of large beam geometry. Opt. Laser Technol..

[23] Lawrence J., Li L. (2000). High power diode laser surface glazing of concrete. J. Laser Appl..

[24] Domone P., Illston J. (2010). Construction Materials: Their Nature and Behaviour.

[25] Alarcon-Ruiz L., Platret G., Massieu E., Ehrlacher A. (2005). The use of thermal analysis in assessing the effect of temperature on a cement paste. Cem. Concr. Res..

